# Predicting the location of the preoptic and anterior hypothalamic region by visualizing the thermoregulatory center on fMRI in craniopharyngioma using cold and warm stimuli

**DOI:** 10.18632/aging.202766

**Published:** 2021-03-26

**Authors:** Jie Kang, Lei Cao, Taoyang Yuan, Lu Jin, Yuanyu Shi, Guofo Ma, Ning Qiao, Chuzhong Li, Yazhuo Zhang, Zhentao Zuo, Songbai Gui

**Affiliations:** 1 Department of Neurosurgery, Beijing Tiantan Hospital, Capital Medical University, Beijing, China; 2 State Key Laboratory of Brain and Cognitive Science, Institute of Biophysics, Chinese Academy of Sciences, Beijing, China; 3 CAS Center for Excellence in Brain Science and Intelligence Technology, Chinese Academy of Sciences, Shanghai, China; 4 Sino-Danish College, University of Chinese Academy of Sciences, Beijing, China; 5 Beijing Neurosurgical Institute, Capital Medical University, Beijing, China

**Keywords:** craniopharyngioma, functional magnetic resonance imaging, hypothalamus, temperature

## Abstract

Hypothalamic nuclei in the preoptic and anterior hypothalamic region (POAH) are critically involved in thermoregulation and neuroendocrine regulation and can be displaced by craniopharyngiomas (CPs). We aimed to locate the POAH by visualizing hypothalamic thermoregulation through task-related functional magnetic resonance imaging (fMRI) to guide hypothalamus protection intraoperatively. Nine adult healthy volunteers (HVs) and thirty-two adult primary CP patients underwent task-related fMRI for POAH localization by warm (60° C) and cold (0° C) cutaneous thermoreceptor stimulation. Approach selection and intraoperative POAH protection were performed based on preoperative POAH localization. In all HVs and patients, significant single positive blood oxygen level-dependent (BOLD) signal changes were located in the POAH. The BOLD activity was significantly greater for cold (P=0.03) and warm (P=0.03) stimuli in patients than in HVs. Gross total resection and near-total resection were achieved in 28 (87.5%) and 4 (12.5%) patients, respectively. New-onset diabetes insipidus and new-onset hypopituitarism occurred in 6 patients (18.8%) and 10 patients (31.3%), respectively. Our findings suggest that cutaneous thermoreceptor stimulation could accurately activate the hypothalamic thermoregulatory center and allow POAH localization through task-related fMRI. Preoperative POAH localization could help neurosurgeons protect hypothalamic function intraoperatively. The CP patients were more sensitive to thermal stimulation.

## INTRODUCTION

Craniopharyngiomas (CPs) are brain tumors with low histological malignant potential that predominantly expand to the hypothalamus [1]. CPs constitute 1.2–4.6% of all intracranial tumors and are managed primarily with surgery [2–4]. The functional hypothalamic nuclei in the preoptic and anterior hypothalamic region (POAH) are critically involved in thermoregulation and neuroendocrine regulation and can be displaced by CPs. Surgical hypothalamic injury may cause severe complications, such as central hyperthermia, hypothalamic obesity, neuroendocrine deficiencies, and neurocognitive deficits, and can result in poor postoperative quality of life [1, 4–6]. A preoperative evaluation of the location of the hypothalamus is critical to avoid surgical hypothalamic injury in patients with CPs [7].

The small size of the hypothalamus still renders locating its nuclei a challenge preoperatively. In recent years, functional magnetic resonance imaging (fMRI) has provided an indirect but non-invasive modality for measuring changes in neuronal activity levels in the brains of awake subjects. Rather than measuring neuronal spiking activity, this technique measures a vascular correlation of neuronal activity and the blood oxygen level-dependent (BOLD) signal [8]. High-resolution fMRI images can be used to measure small changes in blood flow that occur with brain activity [9].

The POAH contains preoptic, supraoptic, and paraventricular nuclei and is critically involved in thermoregulation and neuroendocrine regulation [10]. In this study, we aimed to locate the POAH by stimulating cold and warm cutaneous thermoreceptors using task-related fMRI and evaluate the mode of the functional hypothalamic BOLD response to cold and warm stimuli. Based on the location of the activated POAH in patients with CPs, we could select an appropriate surgical approach preoperatively and protect the structure and function of the hypothalamus intraoperatively.

## RESULTS

### Participant characteristics

From July 2019 to June 2020, 9 HVs and 32 patients with primary CPs participated in this study. The baseline HV characteristics are shown in Table 1. The average patient age was 27.5 (±2.1) years, ranging between 25 and 32 years. The detailed demographic and clinical characteristics of the 32 patients with CPs are summarized in Table 2. In total, 30 patients (93.8%) had vision impairment. Among them, 6 patients had visual acuity loss only, 7 patients had visual field defects only, and 17 patients had both visual acuity loss and visual field defects. Additionally, 21 patients (65.6%) had a headache, 20 patients (62.5%) suffered from endocrine defects, and 4 patients (12.5%) had nausea. All patients underwent surgery with the EEA for CPs in our institution. Overall, 26 patients (81.3%) were diagnosed with adamantinomatous CP, and 6 patients (18.7%) were diagnosed with papillary CP.

**Table 1:** Healthy volunteers characteristics.

**Variables**	**Values**
Age, y	
Median	27
Mean ± SD	27.5 ± 2.1
Sex	
Male	4 (44.4%)
Female	5(55.6%)
Height, m	
Median	1.72
Mean ± SD	1.7 ± 0.1
Weight, kg	
Median	65.0
Mean ± SD	68.1 ± 19.5
BMI, kg/m^2^	
Median	23.1
Mean ± SD	23.8 ± 0.1

**Table 2:** The demographic and clinical characteristics of patients.

**Variable**	**Patients**
Number	32
Sex, no. (%)	
Male	14 (43.8)
Female	18 (56.2)
Age in years	
Median	43.5
Mean ± SD	40.1 ± 14.1
Main complaint, no. (%)	
Vision impairment	30 (93.8)
Visual acuity defect	6 (18.8)
Visual field defect	7 (21.9)
Visual acuity and field defect	17 (53.1)
Headache	21 (65.6)
Endocrine defect	20 (62.5)
Nausea/Vomiting	4 (12.5)
Duration of symptoms in months	
Median	4.5
Mean ± SD	10.8 ± 22.1
BMI	
Median	24.7
Mean ± SD	24.6 ± 4.1

### fMRI results of hypothalamic activity

**Figure 1:**
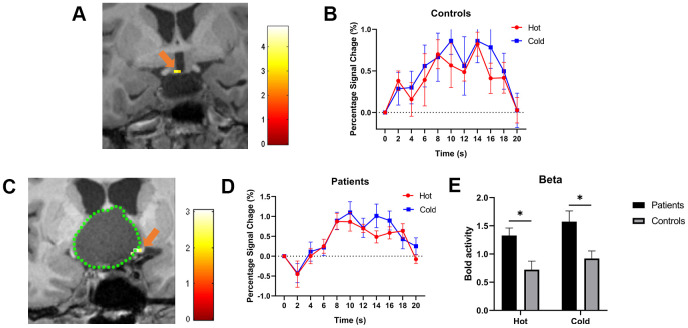
(**A**, **C**) show the activated POAH of the hypothalamus in an HV and a CP patient, respectively. The pseudo color indicates the t-value of the activation strength. The orange arrow indicated the activated POAH in the subject. In Figure 1C, the green dotted line was drawn to mark the boundary of the tumor. (**B**, **D**) show the BOLD responses to cold and warm stimuli in HVs and patients, respectively. (**E**) shows the beta values of warm and cold stimuli between patients and HVs. The BOLD activity in patients was significantly higher than that in HVs in response to both cold and warm stimuli. *: P<0.05. POAH: preoptic and anterior hypothalamic region; HV: healthy volunteer; CP: craniopharyngioma; BOLD: blood oxygen level-dependent.

In all HVs, the significant functional hypothalamic BOLD response area was located in the anterior hypothalamus, and both the cold and warm stimuli produced a significantly positive BOLD signal at the same location. Therefore, the region was speculated to be the POAH (Figure 1A). The time course of activation was similar for both warm and cold stimuli. No significant difference in the BOLD percentage signal change was found between the cold and warm stimuli (P=0.994) (Figure 1B). Based on the BOLD response of the activated position, the location of the POAH was confirmed.

In all patients with CPs, both the cold and warm stimuli also generated a significantly positive BOLD signal, and the significant single functional hypothalamic BOLD signal response around the tumor was distinguished (Figure 1C). Based on the location of the BOLD responses, the POAH was found to be pushed to the anterosuperior (n=5, 15.6%), posterosuperior (n=11, 34.4%), left (n=4, 12.5%), and right (n=12, 37.5%) areas of the tumor (Table 3). The time course of activation was also the same for both warm and cold stimuli. No significant difference in the BOLD percentage change was found between cold and warm stimuli (P=0.895) (Figure 1D).

**Table 3:** Tumour characteristics.

**Patient**	**Lesion volume in cm^3^**	**The location of the tumour**	**The location of POA**	**Tumour resection degree**	**The integrity of pt**	**Pathology**
sub_01	5.3	sup	pos-sup	GTR	com-pt	ACP
sub_02	2.2	int-sup	ant-sup	GTR	com-pt	ACP
sub_03	26.3	sup	right	NTR	non-pt	ACP
sub_04	27.7	sup	pos-sup	GTR	non-pt	ACP
sub_05	6.9	sup	pos-sup	GTR	par-pt	ACP
sub_06	5.1	sup	pos-sup	GTR	par-pt	PCP
sub_07	2.3	sup	pos-sup	GTR	com-pt	ACP
sub_08	39.3	int-sup	pos-sup	GTR	non-pt	ACP
sub_09	1.5	sup	left	GTR	com-pt	ACP
sub_10	25.3	sup	right	GTR	non-pt	PCP
sub_11	2.3	sup	right	GTR	par-pt	ACP
sub_12	16.2	sup	pos-sup	NTR	non-pt	ACP
sub_13	1.6	int-sup	left	GTR	com-pt	ACP
sub_14	3.8	int-sup	left	GTR	par-pt	PCP
sub_15	2.1	sup	right	GTR	com-pt	ACP
sub_16	3.9	int-sup	right	GTR	par-pt	PCP
sub_17	9.4	sup	right	GTR	par-pt	ACP
sub_18	4.3	int-sup	pos-sup	GTR	com-pt	ACP
sub_19	19.1	int-ven	left	GTR	par-pt	ACP
sub_20	6.4	sup	right	GTR	par-pt	PCP
sub_21	13.9	int-sup	ant-sup	GTR	non-pt	ACP
sub_22	3.5	int-ven	ant-sup	GTR	com-pt	ACP
sub_23	6.4	sup	pos-sup	GTR	par-pt	ACP
sub_24	23.6	sup	right	GTR	par-pt	ACP
sub_25	6.6	sup	right	GTR	par-pt	PCP
sub_26	13.9	sup	right	NTR	non-pt	ACP
sub_27	1.7	int-sup	right	GTR	com-pt	ACP
sub_28	19.3	sup	ant-sup	NTR	non-pt	ACP
sub_29	17.1	int-ven	ant-sup	GTR	non-pt	ACP
sub_30	6.9	sup	right	GTR	par-pt	ACP
sub_31	5.6	int-sup	pos-sup	GTR	par-pt	ACP
sub_32	4.7	int-sup	pos-sup	GTR	par-pt	ACP

sup=suprasellar; int-supr=intra-suprasellar; int-ven=intra-third ventricle; pos-sup=posterosuperior; ant-sup= anterosuperior; GTR=gross-total resection; NTR= near-total resection; par-pt= partial pituitary stalk; non-pt=none pituitary stalk; com-pt=complete pituitary stalk; ACP=adamantinomatous craniopharyngioma; PCP=papillary craniopharyngioma.

We compared the beta scores of warm and cold stimuli between patients and HVs. The beta score is the average strength of the BOLD signal caused by stimulation for a period of time. The BOLD activity of the patients was found to be significantly higher than that of the HVs with both cold (P=0.03) and warm (P=0.03) stimuli (Figure 1E).

### Surgical treatment

Different surgical strategies were applied based on the preoperative location of the POAH. The CPC approach was applied in 30 patients, and the EETLT approach combined with the CPC approach was applied in 2 patients. Among all 32 patients, gross total resection (GTR) of the tumor was achieved in 28 patients (87.5%), and near-total resection (NTR) was achieved in 4 patients (12.5%). No significant difference of the rate of GTR was identified among the 4 groups classified by the preoperative location of the POAH (left: 75% [3/4]; right: 91.7% [11/12]; posterosuperior: 90.9% [10/11]; anterosuperior: 80% [4/5]). In our group of cases, the complete pituitary stalk was preserved in 9 patients (28.1%), part of the pituitary stalk was preserved in 14 patients (43.8%), and the pituitary stalk was not preserved in 9 patients (28.1%).

### Complications

Only one patient developed central hyperthermia on the first day after the operation (the highest body temperature was 39.5° C). During the follow-up period, new-onset diabetes insipidus occurred in 6 (18.8%) patients, and new-onset hypopituitarism occurred in 10 (31.3%) patients. Lower incidence rates of new-onset diabetes insipidus and hypopituitarism were found in this study compared to a case summary without the preoperative location of the POAH previously published by our institute (new-onset diabetes insipidus: 18.8% vs 26.4%; new-onset hypopituitarism: 31.3% vs 49.5%) [11].

### Illustrative cases

### Case 19

**Figure 2:**
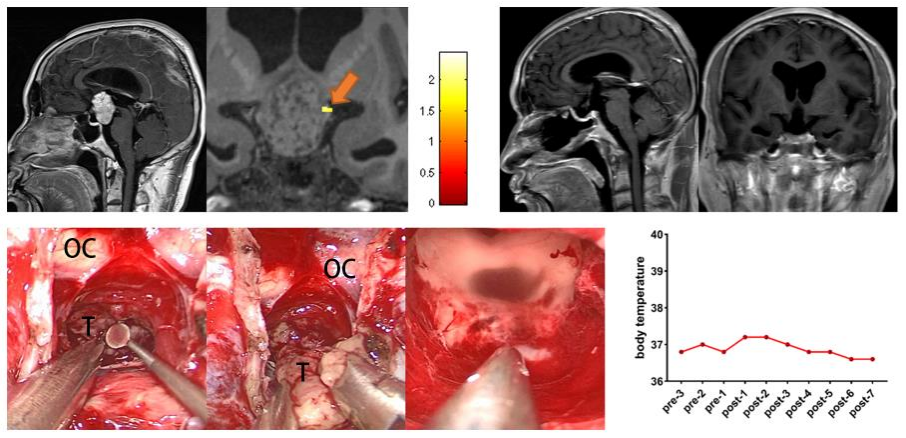
**Preoperative midsagittal contrast-enhanced T1-weighted MRI scans showed a suprasellar solid tumor, and the coronal task-related fMRI showed the activated POAH of the hypothalamus in case 19.** The orange arrow indicated the activated POAH. The postoperative sagittal and coronal contrast-enhanced T1-weighted MRI showed that GTR was achieved. Intraoperative photographs of subject 19 showed that the tumor was not tightly adhered to the surrounding tissues, and the tumor was sharply separated from the surrounding tissues through the CPC approach. After complete resection of the tumor, the midbrain aqueduct and hypothalamus could be observed. No significant increase in body temperature was observed during the perioperative period. fMRI: functional magnetic resonance imaging; POAH: preoptic and anterior hypothalamic region; GTR: gross total resection; CPC: chiasm-pituitary corridor; OC: optic chiasma; T: tumor.

A 48-year-old male patient presented with a 3-month history of progressive blurring of vision and bilateral visual field defects. Preoperative task-related fMRI located the POAH to the left of the tumor. During the operation, the surgeon found that the tumor was not tightly adhered to the surrounding tissues and dissected the tumor via careful microdissection between the tumor and the POAH. Postoperative MRI showed that GTR was achieved. The patient did not present a significant increase in perioperative body temperature. The follow-up period was eight months, and the patient presented no new-onset diabetes insipidus or hypopituitarism (Figure 2).

### Case 3

**Figure 3:**
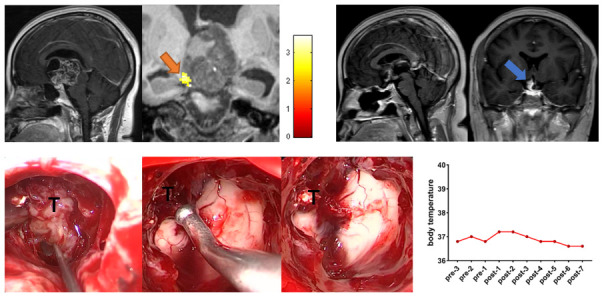
**Preoperative midsagittal contrast-enhanced T1-weighted MRI scans revealed a large solid tumor extending up into the third ventricle, and the coronal task-related fMRI showed the activated POAH of the hypothalamus in case 3.** The orange arrow indicated the activated POAH. The postoperative sagittal and coronal contrast-enhanced T1-weighted MRI showed that NTR was achieved. The blue arrow indicated the tumor that unremoved. Intraoperative photographs showed that the tumor adhered tightly to the surrounding tissues, especially the POAH of the hypothalamus, which was localized preoperatively. To protect the function of the hypothalamus, the part of the tumor that adhered closely to the hypothalamus was not forcibly removed. No significant increase in body temperature was observed during the perioperative period. fMRI: functional magnetic resonance imaging; POAH: preoptic and anterior hypothalamic region; NTR: near-total resection; T: tumor.

A 35-year-old female patient visited the outpatient clinic for a 6-month history of amenorrhea and a 3-month history of progressive blurring of vision and bilateral visual field defects. Preoperative task-related fMRI located the POAH to the right of the tumor. During the operation, the tumor adhered very tightly to the POAH, and the part of the tumor that adhered tightly to the hypothalamus was not forcibly removed. Postoperative coronal postcontrast T1-weighted MRI showed that the retained tumor closely adhered to the POAH. No significant increase in body temperature was observed during the perioperative period. The follow-up period was six months, and the patient presented no new-onset diabetes insipidus or hypopituitarism (Figure 3).

### Case 12

**Figure 4:**
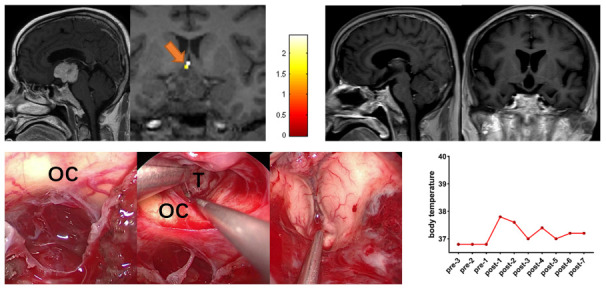
**Preoperative midsagittal contrast-enhanced T1-weighted MRI scans showed a large solid tumor involving the third ventricle, and the coronal task-related fMRI showed the activated POAH of the hypothalamus in case 12.** The orange arrow indicated the activated POAH. The postoperative sagittal and coronal contrast-enhanced T1-weighted MRI showed that GTR was achieved. Intraoperative photographs showed that the tumor was removed through the CPC approach combined with the EETLT approach. After complete resection of the tumor, the midbrain aqueduct and hypothalamus could be observed. No significant increase in body temperature was observed during the perioperative period. fMRI: functional magnetic resonance imaging; POAH: preoptic and anterior hypothalamic region; GTR: gross total resection; CPC: chiasm-pituitary corridor; EETLT: endoscopic endonasal trans-lamina terminalis approach; OC: optic chiasma; T: tumor.

A 57-year-old male patient presented with a 6-month history of visual impairment and headache. Preoperative task-related fMRI located the POAH to the posterosuperior of the tumor. Based on the POAH location identified before surgery, the surgeon removed the tumor through the CPC approach combined with the EETLT approach. Postoperative MR images showed that GTR was achieved. The patient did not present central hyperthermia, diabetes insipidus or hypopituitarism during the follow-up period (9 months) (Figure 4).

## DISCUSSION

In this study, we designed an experiment to locate the POAH using task-related fMRI before surgery in patients with CPs. We found that cold and warm stimuli applied to cutaneous thermoreceptors could activate the POAH and resulted in a significant single positive hypothalamic BOLD response in both HVs and patients with CPs. The BOLD response in patients to both cold and warm stimuli was significantly higher than that in HVs (P=0.03). Identifying the location of the POAH before surgery may help surgeons evaluate the relationship between the CP and the hypothalamus and select an appropriate surgical method to protect the function of the hypothalamus.

An essential factor affecting the postoperative quality of life in CP patients is hypothalamic injury [12]. The hypothalamus is composed of different functional nuclei that release hormones and regulate body temperature. The major nuclei in the POAH include the supraoptic and paraventricular nuclei. Surgical injury to the POAH can cause central hyperthermia, diabetes insipidus and hypopituitarism [13]. The hypothalamus and its nuclei are small, and the signal change in the BOLD response is difficult to image through conventional task-related fMRI [9]. The regulation of the constant body core temperature (TCORE) can be modeled as a reflex, with both feedback and feedforward mechanisms impinging on the integrative circuitry of the POAH [14]. The primary thermoregulatory feedforward sensory signal derives from cold and warm thermoreceptors in the skin. Stimulation of the subcutaneous temperature is strongly influenced by the level of cutaneous blood flow, bringing warm blood from the core into conductive contact with the ambient environment. Stimulation of warm and cold thermoreceptors in the skin activates warm-sensitive neurons in the POAH of the hypothalamus and then activates thermoeffectors and cutaneous vasoconstriction to maintain TCORE [15]. Our experimental design aimed to activate the POAH by stimulating cutaneous thermoreceptors with water at temperatures of 0° C and 60° C and to locate the hypothalamus by task-related fMRI. In this study, when the subjects were stimulated, they remained still for a short time, and the results could accurately reflect the response of the hypothalamus to cold and hot thermal stimulation, through which the position of the hypothalamus could be precisely located. Based on our results, we successfully located and distinguished the POAH in both HVs and patients with CPs. In patients with CPs, the POAH was displaced by the tumor, and the region of the significantly positive BOLD signal response around the tumor was speculated to be the POAH.

The POAH of the hypothalamus plays a crucial role in the regulation of temperature, but the activity of functional hypothalamic responses to cold and warm stimuli in the skin remains controversial [16]. Autonomous thermoregulatory responses to ambient temperature changes, including skin chills caused by cold skin and skin vasodilation caused by skin warmth, transmit thermal sensory signals from the skin to the POAH through the spinoparabrachial pathway [17]. Several studies have reported the use of task-related fMRI to analyze the activity of hypothalamic responses to the consumption temperature and energy (i.e., glucose content) [18, 19]. Jeroen et al. (2005) found a prolonged decrease in the fMRI signal in the hypothalamus following glucose ingestion, and this decrease was dose-dependent [19]. In 2018, Jeroen et al. reported that hypothalamic activity responds similarly to hypothermia and glucose stimulation, and their results suggest that the hypothermia most often associated with the consumption of sugary drinks can lead to a feeling of fullness in the hypothalamus or, more likely, a thirst-quenching response [20]. Although water at low temperatures triggers a hypothalamic response, the temperature was not found to have a stimulatory effect on glucose consumption. In our study, we found an increased BOLD signal immediately after the cold and warm stimuli were applied to the skin, and the BOLD responses of the patients were significantly higher than those of the HVs with both cold and warm stimuli (P=0.03). We speculated that due to compression of the hypothalamus by the tumor, the patients with CPs might have been more sensitive to thermal stimulation, resulting in a stronger response by the hypothalamus.

For CPs with large volumes, especially CPs involving the third ventricle, the fMRI results could help surgeons evaluate the relationship between the tumor and the hypothalamus before treatment. Appropriate awareness of the relationship between these lesions and the hypothalamic nuclei is helpful when selecting an appropriate surgical approach to avoid surgical hypothalamic injury. In this study, the POAH was located anterosuperior to the tumor in 5 patients (15.6%). The EEFLT approach was not used for tumor resection in these patients. In total, 2 patients underwent GTR through the EETLT approach combined with the CPC approach, and the POAH was preoperatively localized to the left of and posterosuperior to the tumor in these two cases. During the operation, based on the preoperative location of the POAH, we sharply dissected the tumor from the hypothalamus with more patience, focusing on the POAH of the hypothalamus.

In our study, only one patient developed central hyperthermia on the first day after the operation (the highest body temperature was 39.5° C), and the average body temperatures of all patients were 37.93±0.77° C over the first three days after the operation and 37.19±0.59° C over 4-7 days after the operation. We did not find any studies that have reported the incidence of central hyperthermia in patients with CPs, so we could not compare our results with those of other studies. However, some studies have reported that central hyperthermia might result in postoperative death [21, 22]. A lower incidence of complications related to hypothalamic injury was also achieved in this study compared to a previous study without the preoperative location of the POAH published by our institute (new-onset diabetes insipidus: 18.8% vs 26.4%; new-onset hypopituitarism: 31.3% vs 49.5%) [11]. The clinical results showed that preoperative localization of the POAH is useful to protect the hypothalamus for tumor resection.

A limitation of our study is that the patients with CPs did not always remain still for a long period during the scan, and only one scan session occurred. If patients can remain still for a long time, several scanning sessions should be implemented to locate the POAH of the hypothalamus more precisely. A second limitation is that temperature stimulation can activate only the anterior hypothalamus; thus, we could judge only the relationship between the tumor and the anterior hypothalamus preoperatively.

## CONCLUSIONS

Thermal stimulation of cutaneous thermoreceptors could activate the POAH on task-related fMRI and resulted in a significant positive BOLD response in the hypothalamus in both HVs and patients with CPs. The patients with CPs were more sensitive to thermal stimulation. Pretreatment localization of the POAH may help surgeons protect the hypothalamus during surgery and reduce the incidence of postoperative complications related to hypothalamic injury.

## MATERIALS AND METHODS

### Participants

Nine healthy adult volunteers (HVs) participated in this study. The individuals with the following conditions were excluded: 1. a history of neurodegenerative, neurodevelopmental, or psychiatric diseases; 2. substance use disorders involving alcohol or heroin; 3. an inability to complete the MRI examinations; and 4. pre-processing issues (i.e., head motion).

A total of thirty-two patients with primary CPs who underwent surgical treatment in the Department of Neurosurgery, Beijing Tiantan Hospital, Capital Medical University, were enrolled in our study. The inclusion criteria for the patient group were as follows: 18-70 years of age; confirmation that the tumor was in the suprasellar and/or third ventricle regions; and histologically proven CPs. The exclusion criteria were as follows: 1. a history of stroke, cerebral trauma, brain surgery or brain radiotherapy, or other intracranial abnormalities (i.e., an arachnoid cyst); 2. an inability to complete the MRI examinations; and 3. pre-processing issues (i.e., head motion).

This study was approved by the Medical Ethics Committee of Beijing Tiantan Hospital. Written informed consent was obtained from the legal representatives of all the patients and all the HVs in accordance with the Declaration of Helsinki.

### Experimental procedure

**Figure 5:**
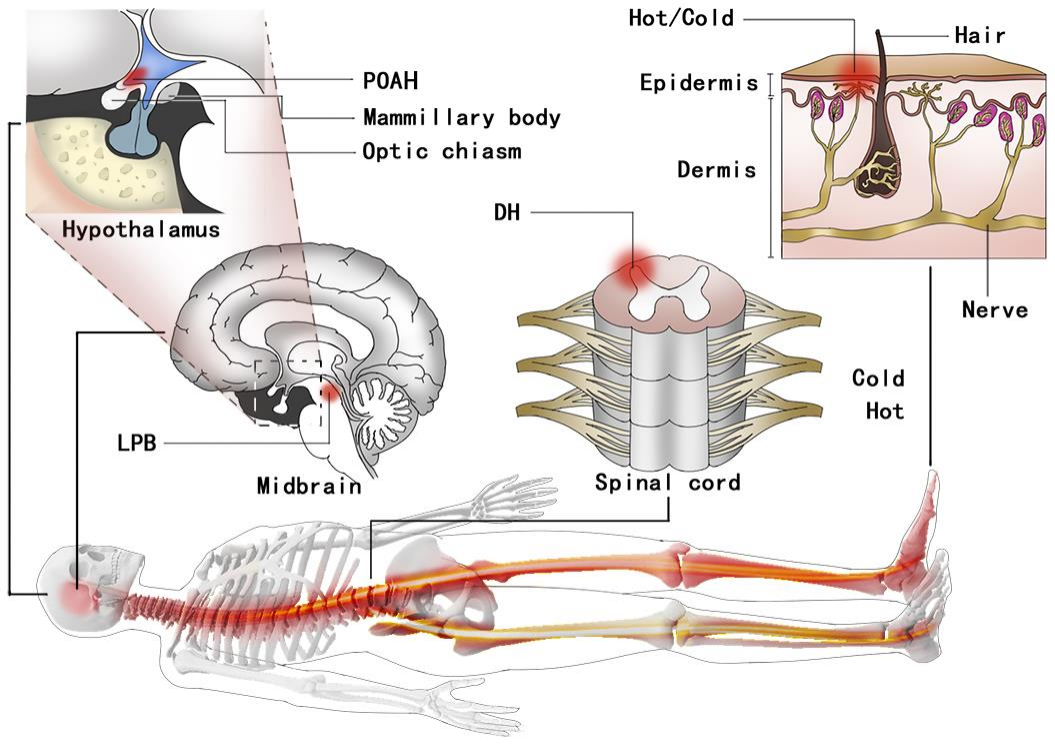
**A schematic of the feedback regulation mechanism of the TCORE circuit integrating the POAH.** TCORE feedback sensory signals come from thermoreceptors in the skin. Then, the signals are transmitted to the DH. Cutaneous thermoreceptor signals from the DH to the POAH that drive feedforward thermoregulatory responses are integrated with a variety of currently unidentified inputs in the LPB of the pons. TCORE: constant body core temperature; POAH: preoptic and anterior hypothalamic region; DH: dorsal horn; LPB: lateral parabrachial nucleus.

The hypothalamus is a small area in the brain involved in releasing hormones and regulating body temperature [23]. The POAH of the hypothalamus is the center of feedforward regulation of reflex thermoregulation [24], contains numerous thermosensitive neurons and receives feedforward thermosensory signals from cutaneous thermoreceptors [25]. Primary cold and warm cutaneous thermoreceptors synapse on somatosensory dorsal horn (DH) neurons in the spinal cord and send projections to the pontine lateral parabrachial nucleus (LPB) [26–28]. The cutaneous thermoreceptor signaling from the LPB to the POAH drives feedforward thermoregulatory responses (Figure 5) [29, 30].

**Figure 6:**
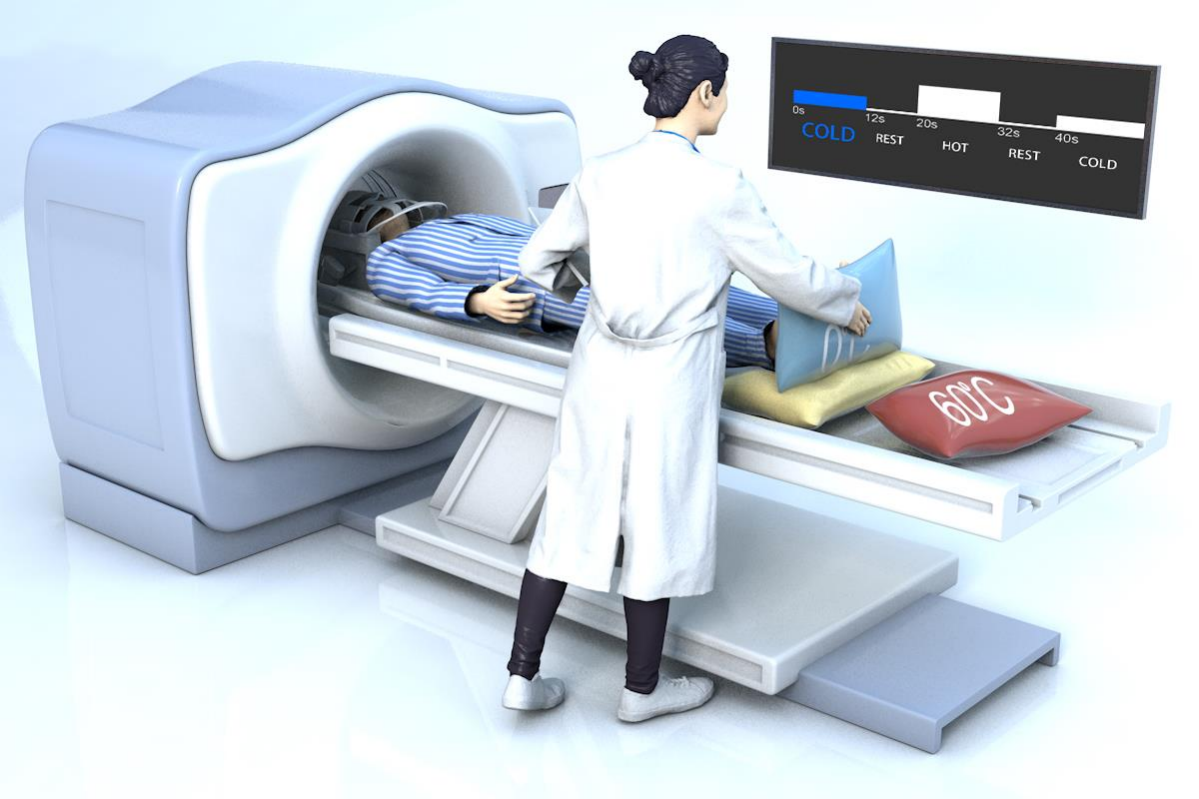
**A schematic of the experimental operation.** The subject was lying supine in the scanner in the 64-channel head coil. The researcher administered a balanced-design stimulus according to task prompts. A countdown prompted the operator to start and end the stimulus five seconds before the end of each block.

An ice-water mixture at 0° C (cold stimulus) and a warm compress bag at 60° C (warm stimulus) were used to stimulate cold and warm cutaneous thermoreceptors. A balanced-design stimulus was administered during the fMRI scan by an independent person at the State Key Laboratory of Brain and Cognitive Science, Institute of Biophysics, Chinese Academy of Sciences, according to task prompts. Task-related fMRI included ten cold blocks (ice-water mixture at 0° C) and ten warm blocks (warm compress bag at 60° C) interleaved with rest blocks. The cold and warm blocks lasted 12 s, and the rest block lasted 8 s. A countdown prompted the operator to start and end the stimulus five seconds before the end of each block (Figure 6). The participant was in the supine position in the scanner in the 64-channel head coil.

### MRI data acquisition and analysis

All subjects were scanned on a 3.0-T Siemens scanner (Siemens Healthineers, Erlange, Germany) at the Beijing MRI Center for Brain Research, Institute of Biophysics, Chinese Academy of Sciences, with a 64-channel head coil. The T1-weighted images were obtained with a 3D sagittal magnetization prepared rapid acquisition gradient echo (MPRAGE) sequence (resolution = 0.8 mm isotropic voxels; field of view = 256 × 240 mm^2^; slices = 224; flip angle = 8°; TI/TR/TE = 1000/2400/2.22 ms; data matrix= 320 × 300; bandwidth = 220 Hz/px; iPAT = 2). A gradient echo planar imaging sequence was used to acquire the task-related fMRI data covering the whole thalamus (resolution = 1.4 mm isotropic voxels; field of view = 192 × 192 mm2; slices = 50; flip angle = 90°, TR/TE = 2000/31 ms; iPAT = 2).

BOLD fMRI data were pre-processed using the Statistical Parametric Mapping toolbox (SPM12, http://www.fil.ion.ucl.ac.uk/spm) in the MATLAB environment (MATHWORKS, CA, United States). The pre-processing procedure included slice timing, motion correction, co-registering fMRI images to T1-weighted images, and smoothing. Then, a general linear model was used to analyze the activation strength of each stimulus.

In HVs, based on T1-weighted images, the activated functional BOLD response in the hypothalamus was used as the region of interest (ROI) to observe the functional POAH response to the cold and warm stimuli. In patients, the activated functional BOLD response around the tumor was used as the ROI to locate the POAH and observe the functional POAH response to the thermal stimuli.

### Surgical strategies based on the location of the POAH

The endoscopic endonasal approach (EEA) was applied in all patients. Based on the location of the activated POAH, we divided the POAH position into four types according to the location of the POAH relative to the tumor: left, right, posterosuperior and anterosuperior. The tumor was first debulked to depress the tumor capsule. Once the tumor was debulked and decompressed, the surgeon dissected the extracapsular tumor away from the hypothalamus via careful microdissection based on the preoperative location of the POAH on fMRI. We sharply dissected the tumor from the hypothalamus under direct vision of the interface between the tumor and hypothalamus to avoid hypothalamic injury. For large tumors involving the third ventricle, if the POAH was located anterosuperior to the tumor, the endoscopic endonasal trans-lamina terminalis (EETLT) approach was not used to remove the tumor to protect the POAH, and if the POAH was not located anterosuperior to the tumor, the EETLT approach combined with or without the chiasm-pituitary corridor (CPC) approach could be considered for tumor removal.

Gross total resection (GTR) was defined as resection without visible remnant tumor according to an intraoperative assessment and postoperative MRI. The perioperative body temperature, extent of resection and endocrine outcomes were collected to evaluate the significance of this method.

### Statistical analysis

Uncorrected P values <0.05 were deemed significant for all tests. The threshold was increased to distinguish the single activated area around the tumor to locate the POAH in patients with CPs. All fMRI results are reported as the percentage of BOLD signal changes relative to the BOLD signal at the beginning of each stimulus. For presentation purposes, data were pooled into "second" data points, and the start of each stimulus was considered the baseline BOLD response. Unpaired samples were analyzed using the Mann-Whitney U test or the Kruskal-Wallis test, while paired samples were assessed using the Wilcoxon t test. Differences correlating to an error of probability with P< 0.05 were considered statistically significant. Two-way ANOVA was used to determine whether significant differences existed between cold and warm stimuli and in BOLD values between the HVs and patients.
